# Variant G6PD levels promote tumor cell proliferation or apoptosis via the STAT3/5 pathway in the human melanoma xenograft mouse model

**DOI:** 10.1186/1471-2407-13-251

**Published:** 2013-05-22

**Authors:** Tao Hu, Chunhua Zhang, Qiongling Tang, Yanan Su, Bo Li, Long Chen, Zheng Zhang, Tianchi Cai, Yuechun Zhu

**Affiliations:** 1 Department of Biochemistry and Molecular Biology, Kunming Medical University, Kunming 650031, China; 2 Department of laboratory medicine, The Third People’s Hospital of Yunnan Province, Kunming, China; 3 Yunnan Provincial Maternal and Child Health Hospital, Kunming, China; 4 Shenzhen Luohu Maternal and Child Health Hospital, Shenzhen, China; 5 GenProMarkers, Inc., 9700 Great Seneca Highway, Suite182, Rockville, MD 20850, USA; 6 Department of laboratory animal, Kunming Medical University, Kunming, China

**Keywords:** Glucose-6-phosphate dehydrogenase, Melanoma, Nude mice, Apoptosis, Signal transduction and transcription activator

## Abstract

**Background:**

Glucose-6-phosphate dehydrogenase (G6PD), elevated in tumor cells, catalyzes the first reaction in the pentose-phosphate pathway. The regulation mechanism of G6PD and pathological change in human melanoma growth remains unknown.

**Methods:**

HEM (human epidermal melanocyte) cells and human melanoma cells with the wild-type *G6PD* gene (A375-WT), G6PD deficiency (A375-G6PD∆), G6PD cDNA overexpression (A375-G6PD∆-G6PD-WT), and mutant G6PD cDNA (A375-G6PD∆-G6PD-G487A) were subcutaneously injected into 5 groups of nude mice. Expressions of G6PD, STAT3, STAT5, cell cycle-related proteins, and apoptotic proteins as well as mechanistic exploration of STAT3/STAT5 were determined by quantitative real-time PCR (qRT-PCR), immunohistochemistry and western blot.

**Results:**

Delayed formation and slowed growth were apparent in A375-G6PD∆ cells, compared to A375-WT cells. Significantly decreased G6PD expression and activity were observed in tumor tissues induced by A375-G6PD∆, along with down-regulated cell cycle proteins cyclin D1, cyclin E, p53, and S100A4. Apoptosis-inhibited factors Bcl-2 and Bcl-xl were up-regulated; however, apoptosis factor Fas was down-regulated, compared to A375-WT cells. Moderate protein expressions were observed in A375-G6PD∆-G6PD-WT and A375-G6PD∆-G6PD-G487A cells.

**Conclusions:**

G6PD may regulate apoptosis and expression of cell cycle-related proteins through phosphorylation of transcription factors STAT3 and STAT5, thus mediating formation and growth of human melanoma cells. Further study will, however, be required to determine potential clinical applications.

## Background

Glucose-6-phosphate dehydrogenase (G6PD) is a critical enzyme in mammalian erythrocytes that catalyzes the first reaction in the pentose-phosphate pathway [1]. G6PD abnormalities affect as much as 7.5% of the global population, with a wide range of occurrence based on geographic distribution. These abnormalities occur in as little of 0.1% of certain Japanese populations to more than 35% in some African and European populations [2]. In fact, G6PD was the first prevalent enzyme deficiency to be characterized by the World Health Organization (WHO) in 1984 [3]. Around the world, between 140 and 160 distinct G6PD mutations have been reported [1].

In China, at least 24 mutations have been detected in the *G6PD* gene [4,5]. Our previous study showed that *G6PD* Mahidol (487G>A) was the most common *G6PD* variant in the Achang ethnic group of Yunnan Province [6]. Otherwise, G6PD Mahidol is a common deficient variant caused by a (163)glycine-serine mutation that occurs in about 15% of individuals in populations across Southeast Asia [7,8]. The prevalence of this mutation can be accounted for by strong positive selection over the past 1500 years that occurred in response to certain parasites, including malaria-causing agents such as *Plasmodium vivax* and *Plasmodium falciparum*, that target humans [9]. Like many mutations involved in parasitic resistance, G6PD Mahidol is a relatively recent polymorphism generally referred to as a ‘loss-of-function’ mutation, occurring via a single-nucleotide change and ultimately resulting in reduced G6PD production [10]. While G6PD Mahidol may confer a selective advantage to parasitic infections, such as malaria, this genetic variation may also have other detrimental affects to the immune response and may be implicated in the cell cycle of abnormal or cancerous cells [9,10].

It has been found that the expression or enzyme activity of G6PD is elevated in multiple tumors, including leukemia [11], gastrointestinal cancers [12], renal cell carcinomas [13], colon cancers [14], breast cancers [15], endometrial carcinomas [16], prostate and liver cancer [17,18]. Considering that ectopic G6PD expression increases the levels of nicotinamide adenosine dinucleotide phosphate (NAPDH) and glutathione, G6PD may promote the survival of tumor cells through maintenance of both extracellular pH and redox potential [19,20], though little is known about how G6PD regulates cell proliferation and apoptosis in tumors.

Persistent expression and excessive activation of STAT3 and STAT5 is apparent in melanoma cells. Accordingly, STAT3 and STAT5 have become interesting potential target molecules for the treatment of melanoma [21,22]. Our previous *in vitro* study demonstrated a significant reduction in the P-STAT5/STAT5 ratio of A375-G6PD∆ cells following knockdown of G6PD in A375 cells, while the P-STAT5/STAT5 ratio significantly increased following overexpression of G6PD in the G6PD-knockdown A375 cells. This suggested that G6PD promotes the proliferation of A375 cells and is associated with induction or activation of STAT5. In addition, it has been found that STAT3 is persistently activated, and P-STAT3 expression is significantly elevated in A375 cells. STAT3 expression increased by five-fold in G6PD-knockdown A375 cells compared to normal A375 cells, and P-STAT3 expression levels in G6PD-knockdown A375 cells was 20% of that in A375-WT cells (unpublished data). The activation of STAT3 is fast and transient under normal physiological conditions, and it is strictly regulated [23]. These findings indicate that STAT3 and STAT5 play important roles in mediating the biological characteristics of melanomas. However, the underlying mechanism remains unclear.

The current study further explores the relationship and mechanism of action of G6PD and melanoma cell proliferation and apoptosis using a mouse model of tumor formation. Human dermal melanoma cells expressing the wild-type *G6PD* gene (A375-WT), G6PD-deficient A375 cells (A375-G6PD∆), and A375-G6PD∆ cells with overexpression of normal G6PD cDNA (A375-G6PD∆-G6PD-WT) and mutant G6PD cDNA (A375-G6PD∆-G6PD-G487A) were administered to mice in order to compare the time of initial tumor formation, tumor size, and pathological changes. In addition, the expression of G6PD and its activity, cell cycle-related proteins, apoptosis-related proteins, and STAT3/STAT5 in tumor tissues were determined in order to provide full documentation of the regulatory mechanisms involved with *in vivo* melanoma growth associated with G6PD.

## Methods

### Cell culture

Human melanoma cell lines (A375) with knocked down *G6PD* genes (A375-G6PD∆) were established from wild-type human dermal melanoma cell lines (A375-WT) (Cell Bank of the Chinese Academy of Sciences) as previously described [24]. Wild-type and mutant-type G6PD genes (G487A,G→A) were amplified using PCR and then cloned into a retroviral vector (pBABEpuro) to yield the expression vectors pBABEpuro-G6PDWT and pBABE-puro-G6PDG487A, respectively. The expression vectors were transfected into 293FT package cells (R70007, Invitrogen, USA) using a retrovirus packaging kit (D6161, Takara, Japan) to produce recombinant viruses. The recombinant retrovirus was used to infect the A375-G6PD∆ cells and was subsequently screened for 7 days using puromycin (0.5 μg/mL) (J593, Amreso, USA). Then, clones positive for puromycin-resistance were co-cultured in G418 (200 μg/mL) and puromycin (0.25 μg/mL) to yield A375∆-WT and A375∆-G6PDA cells exhibiting overexpression of *G6PD*. The passage and digestion of cells were similar to those of the A375-G6PD∆ cells. Normal human epidermal melanocytes (HEM) were isolated from primary-cultured melanocytes from the foreskin of healthy children [25] as a source for mouse xenografts.

### Establishment of a melanoma xenograft nude mouse model

A total of 25 4–5 w BALB/c strain nude mice (18–20 g) (Beijing HFK Bioscience Co, Ltd, Beijing China) (Animal license number: SCXK 2009–0004; Beijing) were housed and raised in the laboratory animal center of the Affiliated Cancer Hospital of Sun Yat-sen University. The treatment and use of animals during the study was approved by the Animal Ethics Committee of Sun Yat-sen University.

Mice were randomly assigned to 5 groups of 5 mice each: normal human epidermal melanocytes (HEM), human dermal melanoma cells (A375-WT), G6PD-deficient A375 cells (A375-G6PD∆), A375-G6PD∆ cells with overexpression of normal G6PD cDNA (A375-G6PD∆-G6PD-WT), and A375-G6PD∆ cells with overexpression of mutant G6PD cDNA (A375-G6PD∆-G6PD-G487A). Cells in the log-phase stage of growth were harvested and digested into single cells using 0.25% pancreatin. Cells were washed with Dulbecco’s Modified Eagle Medium (DMEM) without serum. A volume of 1 ml of each cell type (1.5×10^6^/ml) was injected intradermally into the left axilla of the mice subjects. After seeding, liquid absorption at the injection site, tumor growth (volume and weight), and mouse survival were observed. Tumor volume was measured on days 5, 7, 12, 16, 19, 21, and 23 post-injection. On day 23, all mice were sacrificed, and tumors were isolated for determination of weight and volume.

The largest (*a*) and smallest diameters (*b*) of each tumor were measured twice on days 5, 7, 12, 16, 19, 21, and 23 to estimate tumor volume (*V*) using the formula V = 0.52 × *a*^2^ × b [26]. Mean tumor volumes were used to plot tumor growth curves for each group of mice.

### Immunohistochemistry

Immunohistochemistry was performed to determine associations between the G6PD expression in various experimental groups, tumor growth, and pathological changes.

Tumor samples were fixed in 4% formaldehyde, embedded in paraffin wax, and then cut into 4 μm sections using a microtome. The sections were stained with hematoxylin and eosin (HE).

Fixed tumor samples were prepared into 30 μm frozen sections and incubated with 2% goat serum at 37°C for 20 min, followed by incubation with rabbit-anti-G6PD (1:500, Boster, China) at 4°C overnight. After washing with PBS, the sections were incubated with horseradish peroxidase (HRP)-conjugated goat anti-rabbit IgG (HRP-IgG) at 37°C for 30 min and colored with 3,3′-Diaminobenzidine (DAB) at room temperature. PBS was substituted for the rabbit-anti-G6PD antibody in negative control subjects. The number of cells positive for G6PD was then calculated.

### Determination of G6PD activity

Tumor samples (20 mg) were homogenized with 5 ml of PBS at 4°C and centrifuged at 5000 r/min for 3 min. The supernatant (30 μl) was treated with a G6PD Activity Assay Kit reagent (BioVision, USA) according to the manufacturer’s instructions. The optical density (OD value, A1) of the positive control supplied by the kit was measured at 450 nm using a U-1800 ultraviolet spectrophotometer (Hitachi, Japan). After 30 min at 37°C the OD value (A2) was measured a second time. The kit’s standard reagent, nicotinamide adenine dinucleotide hydride (NADPH), was diluted 3 times, and the standard curve was plotted. *A*1 and *A*2 were introduced into the standard curve to generate the total NADH (*B*). The G6PD activity was then calculated using the formula: G6PD activity (mU/ml) = *B*/30 × *V* × dilution times.

### Extraction of total RNA, reverse transcription, and quantitative real-time PCR

Quantitative real-time PCR (qRT-PCR) was used to quantify the expression of *G6PD* mRNA in the experimental groups. Tumor samples (60 mg) were ground under liquid nitrogen, lysed with 1 ml of Trizol (Takara, Japan), and total RNA was extracted using Trizol (Invitrogen, USA). Total RNA (2 μg) was added to the tumor extract with Moloney Murine Leukemia Virus Reverse Transcriptase (MMLV-RT, Takara, Japan) to synthesize cDNA, and the reverse transcript was used as the template for qRT-PCR using a Tower qRT-PCR system (Analytic Jena, Germany). The qRT-PCR was conducted using 2×Mix SYBR Green I (Biosea, USA) (10 μl), primer (0.25 μl, 10 pmol/L), template DNA (1 μl), and sterile water (8.5 μl). All PCR reactions included initial denaturation and multiple cycles at (95°C for 3 min); 39 cycles at 95°C for 10 s, 55°C for 10 s, and 72°C for 30 s; followed by 95°C for 10 s, 65°C for 5 s, and a final 95°C for 15 s. The primer for each gene was synthesized by Invitrogen (USA), and the sequences were determined (Table 1).

**Table 1:** Real-time PCR primer sequences

**Gene**	**Primer**	**Sequence of primer**	**Length of sequence (Base)**	**Length of amplified product (bp)**
*G6PD*	G6PD-F	5′TGAGCCAGATAGGCTGGAA3′	19	225
	G6PD-R	5′TAACGCAGGCGATGTTGTC3′	19	
*CyclinD1*	CyclinD1-F	5′CGGTAGTAGGACAGGAAGTT3′	20	120
	CyclinD1-R	5′CTGTGCCACAGATGTGAAGT3′	20	
*Fas*	Fas-F	5′GCCACCGACTTTAAGTTTGC3′	20	102
	Fas-R	5′CGAGCTCACTTCCTCATCCT3′	20	
*STAT3*	STAT3-F	5′CACCCGCAATGATTACAGTG3′	20	126
	STAT3-R	5′CGGTCTGACCTCTTAATTCG3′	20	
*STAT5*	STAT5-F	5′CACCCGCAATGATTACAGTG3′	20	126
	STAT5-R	5′CGGTCTGACCTCTTAATTCG3′	20	
*β-actin*	β-actin-F	5′TGGCACCCAGCACAATGAA3′	20	186
	β-actin-R	5′CTAAGTCATAGTCCGCCTAGAAGCA3′	25	

### Western blot

Tumor samples were lysed for 30 min in CytoBuster Protein Extraction Buffer (Novagen, USA) and centrifuged at 12000 rpm. The supernatant was collected, total protein was measured, and 50 μg was used for 10% sodium dodecyl sulfate polyacrylamide gel electrophoresis (SDS-PAGE). The protein was then transferred to a nitrocellulose (NC) membrane and sealed with Tris-Buffered Saline Tween-20 (TBST) containing 5% non-fat milk powder. The membrane was subsequently incubated with rabbit anti-human STAT3, P-STAT3, STAT5, and P-STAT5 proteins and mouse anti-human β-actin (1:500 to 1:1000, Boster) at 4°C overnight. After washing in TBST, the membrane was incubated with HPR conjugated secondary antibodies (1:6000) at 25°C, and the protein quantity was determined using electrochemiluminescence (ECL) technique (BestBio, USA). The results were photographed using the JS Gel Imaging System (Peiqing, China) and the grey density was calculated using SensiAnsys software (Peiqing, China).

### Statistical analysis

Statistical analyses were performed using SPSS statistical software version 13.0 (IBM, USA). One-way analysis of variance (ANOVA) with 5 levels was used with a completely randomized design, and the homogeneity of variance was tested. A *q* test (Student-Newman-Keuls) was used to compare the differences between groups, and a rank sum test was performed to randomly compare treatments. A *P*-value of less than 0.05 was considered statistically significant (*P* < 0.05). Values were expressed as means ± standard deviation (mean ± SD).

## Results

### Construction and identification of cell lines

**Figure 1:**
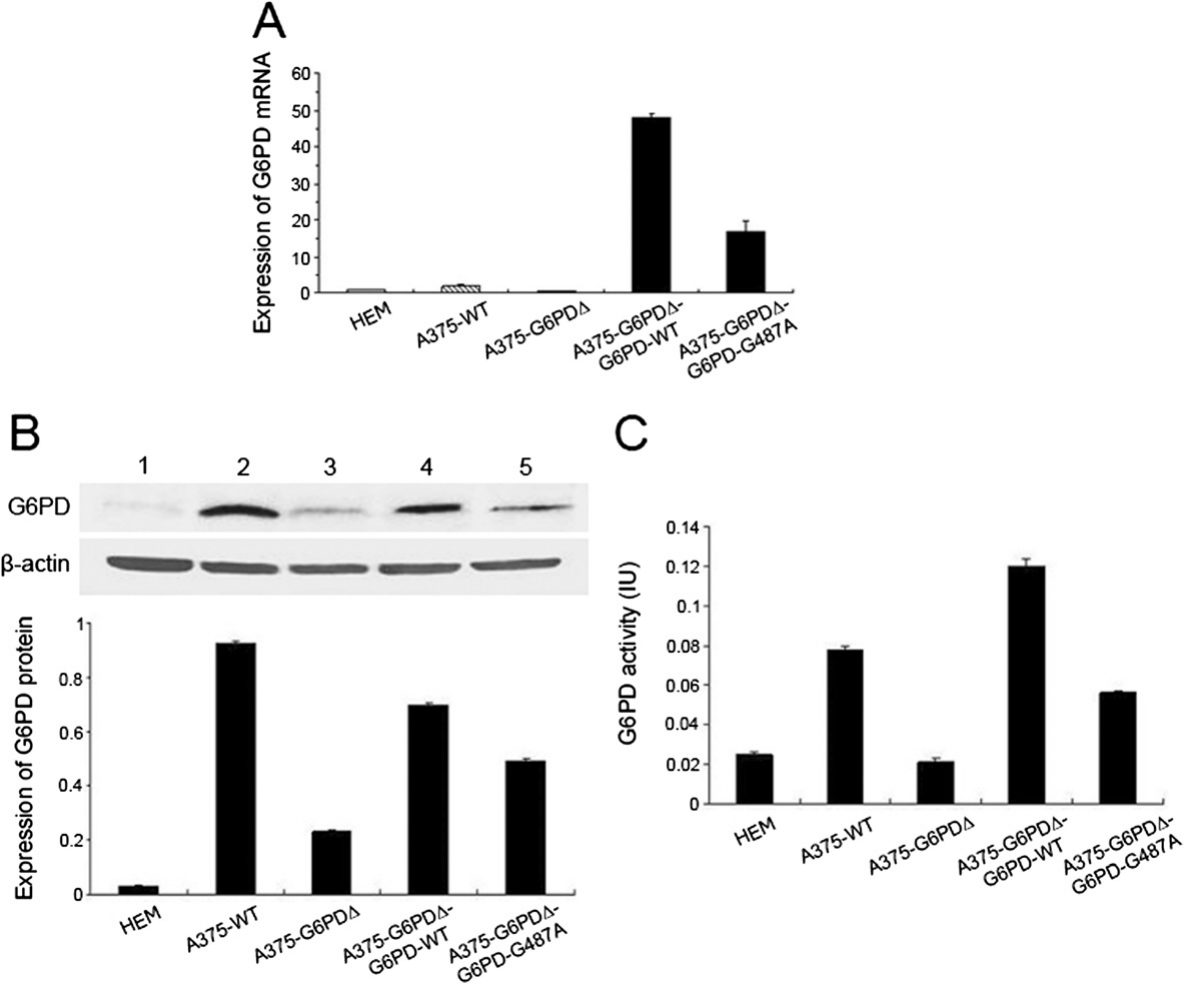
**Identification of tumor cell lines. A**, Determination of mRNA expression of G6PD in various cell lines; **B**, Determination of expression of G6PD protein in various cell lines; **C**, Determination of G6PD activity in various cell lines.

After multiple freeze-thaw cycles (≥ 20), fluorescent protein [24] was stably expressed in A375-G6PD∆, A375-G6PD∆-G6PD-WT, and A375-G6PD∆-G6PD-G487A cells, which were morphologically normal (data not shown). The expression of G6PD mRNA, protein quantity, and G6PD activity were all higher in A375 cells than those in normal HEM cells. After *G6PD* knockdown, the expression of *G6PD* mRNA, G6PD protein, and enzyme activity were significantly down-regulated (*P* < 0.01). In contrast, the G6PD mRNA, protein expression, and enzyme activity were correspondingly elevated after the normal or *G6PD* deficient gene was introduced into A375-G6PD∆ cells. A375-G6PD∆, A375-G6PD∆-G6PD-WT, and A375-G6PD∆-G6PD-G487A cell lines were successfully established (Figure 1).

### Effect of G6PD deficiency on tumor formation in nude mice injected with A375 cell lines

**Figure 2:**
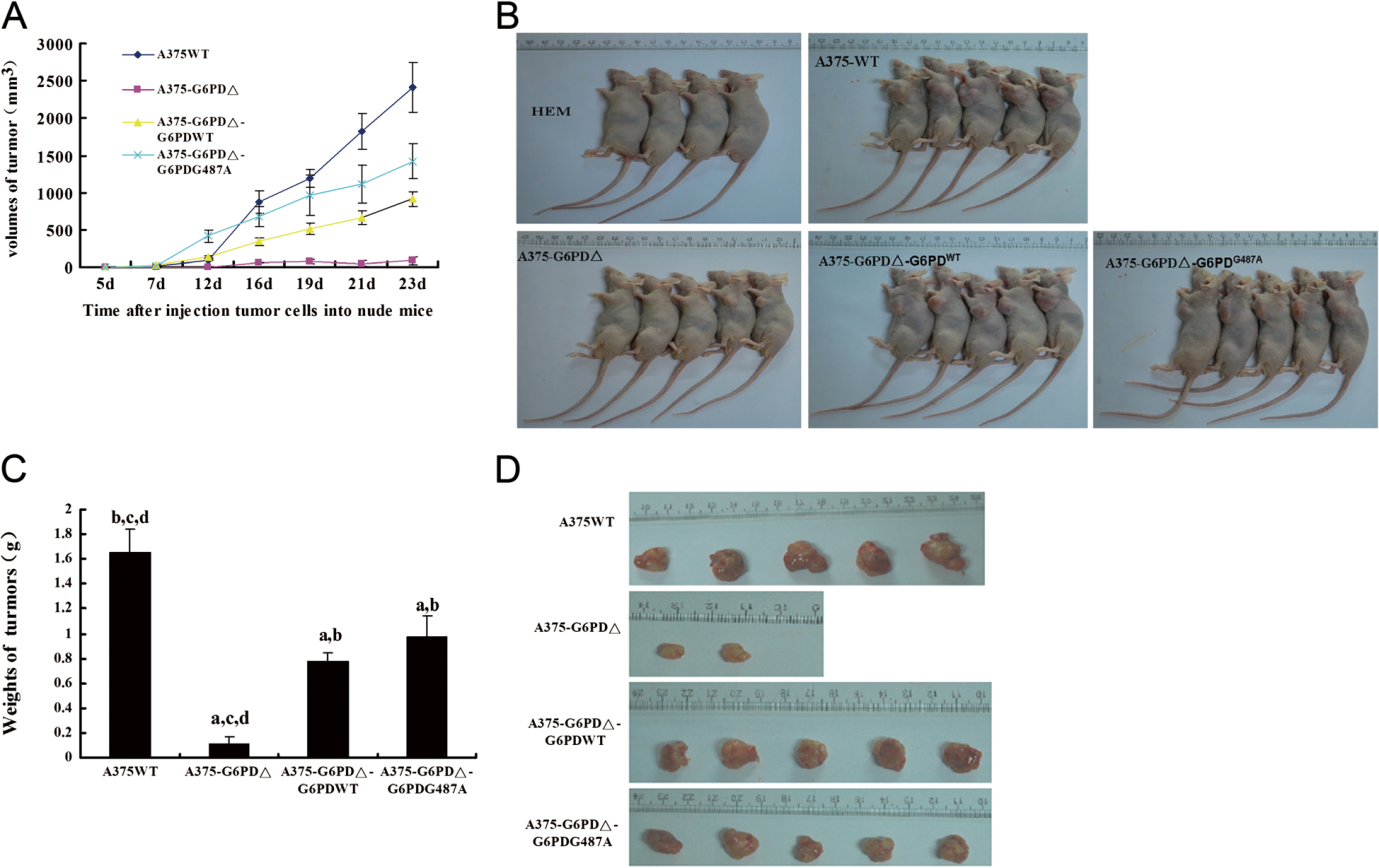
**Tumor formation and growth of 4 tumor cells in nude mice.** After normal human epidermal melanoma cells (HEM), human dermal melanoma cells (A375-WT), G6PD-deficient A375 cells (A375-G6PD∆), A375-G6PD∆ cells with overexpression of normal G6PD cDNA (A375-G6PD∆-G6PD-WT) and A375-G6PD∆ cells with overexpression of mutant G6PD cDNA (A375-G6PD∆-G6PD-G487A) were injected into the nude mice. The tumor size was measured on days 7, 12, 16, 19, 21 and 23 post-injection, and the tumor volume growth curve **(A)** was plotted. The nude mice were photographed on day 23 **(B)**, and then sacrificed. The tumors were isolated, and the weights **(C)** and volume of the tumors were measured **(D)**.

Tumor formation was observed in 2 of 5 mice injected with A375-G6PD∆ cells. These tumors were the last to form and grew the slowest among the treatment groups. The fastest tumor growth was observed in the A375-WT cell group (Figure 2). Tumor formation was observed 12 d after injection of cells with G6PD deficiency. Tumor sizes observed in the G6PD deficiency group (A375-G6PDΔ) were significantly smaller than those observed in the other groups at 12 to 23 d post-injection (*P* < 0.05). Tumors presence was observed at 7 d post-injection in A375-WT cells, and tumor size was significantly greater in this group than in other groups at 16 days post-injection (*P* < 0.05).

The smallest tumors at 23 d were observed in the G6PD deficiency group (*P* < 0.05), while the largest tumors were in the A375-WT cell group (*P* < 0.05). There was no significant difference in tumor weights between the A375-G6PD∆-G6PD-G487A and A375-G6PD∆-G6PD-WT cell groups (*P* > 0.05), but both exhibited tumors that were larger than those observed in the G6PD deficiency group (*P* < 0.05, Figure 2C).

Pathological staining results showed that tumors in the A375-WT cell group appeared to be more malignant than those observed in other groups. Tumor cells taken from the A375-G6PDΔ group exhibited the most benign changes compared with other groups (Additional file 1: Table S1 and Additional file 2: Figure S1). No metastasis was observed in either the liver or lungs in any groups, as determined via microscopic techniques.

### G6PD expression and enzyme activity in nude mice tumor tissues

**Figure 3:**
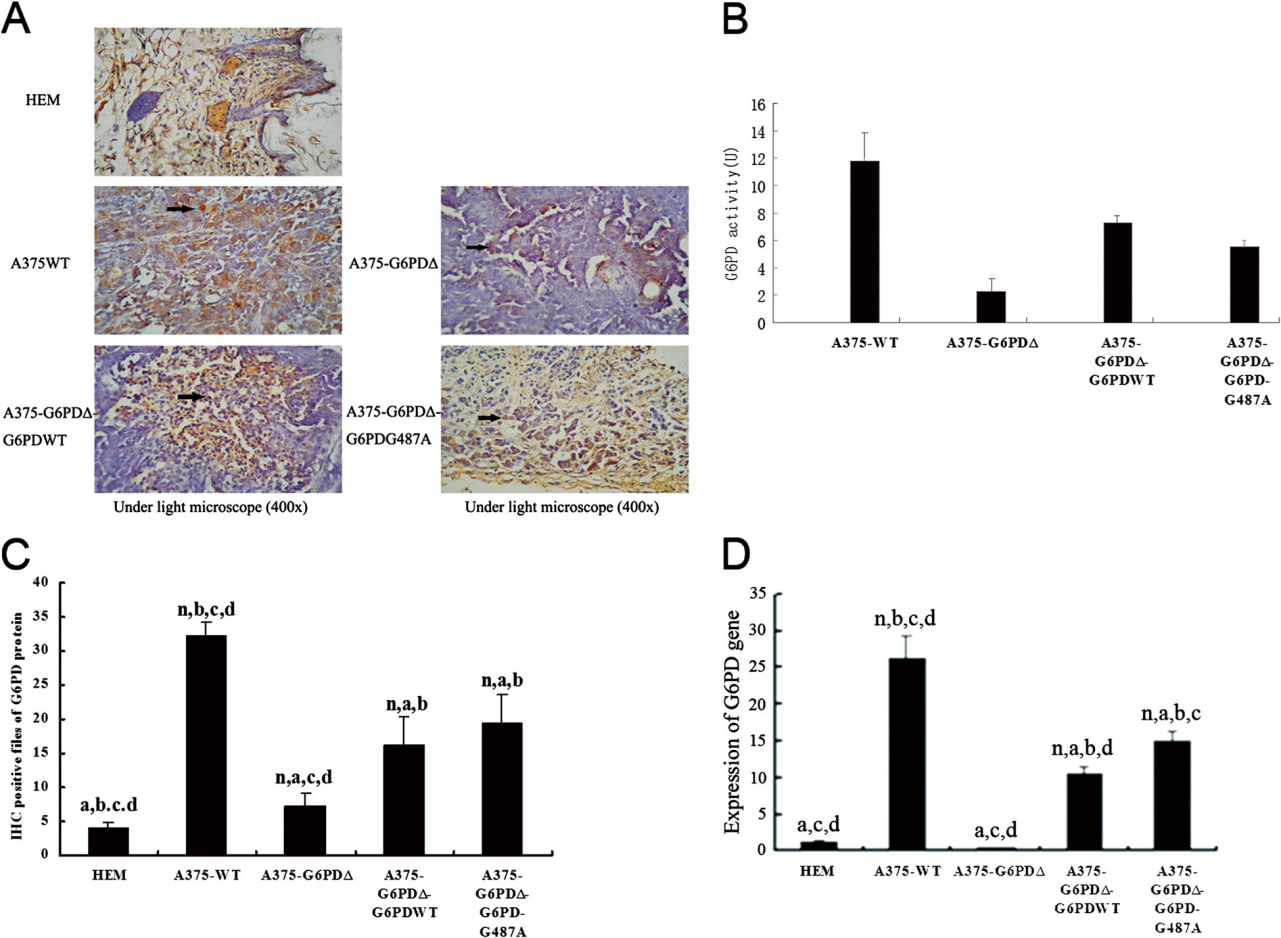
**G6PD protein and mRNA expression and activity in tumor tissues of nude mice from different experimental groups. A**, Paraffin-embedded sections of the tumor tissues of nude mice from different groups were made and the G6PD expression is determined using immunohistochemistry (400×); **B**, Immunohistochemistry reveals the G6PD-positive cells in tumor tissues; **C**, Changes of G6PD activity. n, a, b, c and d indicate statistically significant differences, *P* < 0.05. n, vs. HEM cells group; a, vs. A375-WT cells group; b, vs. A375-G6PDΔ cells group; c, vs. A375-G6PDΔ-G6PDWT cells group; d, vs. A375- G6PDΔ-G6PD-G487A cells group. **D**, mRNA expression of G6PD detected by qRT-PCR.

Immunohistochemistry demonstrated that the largest number of G6PD-positive cells were present in the A375-WT cell group, while the amount of G6PD-positive cells was significantly reduced in the A375-G6PD∆ cell group (Figure 3A, 3C). Similarly, qRT-PCR indicated that the level of *G6PD* mRNA detected in the A375-WT cell group increased by 66-fold compared with that observed in the A375-G6PD∆ group (*P* < 0.05, Figure 3D). The highest G6PD activity was observed in the A375-WT cell group, followed consecutively lower activities observed in the A375-G6PD∆-G6PD-WT and A375-G6PD∆-G6PD-G487A cell groups. The lowest G6PD activity was observed in the A375-G6PD∆ cell group (*P* < 0.01, Figure 3B), demonstrating the association of G6PD with melanoma formation and growth in nude mice.

### Correlation of G6PD in tumor tissues with cell cycle protein expression

**Figure 4:**
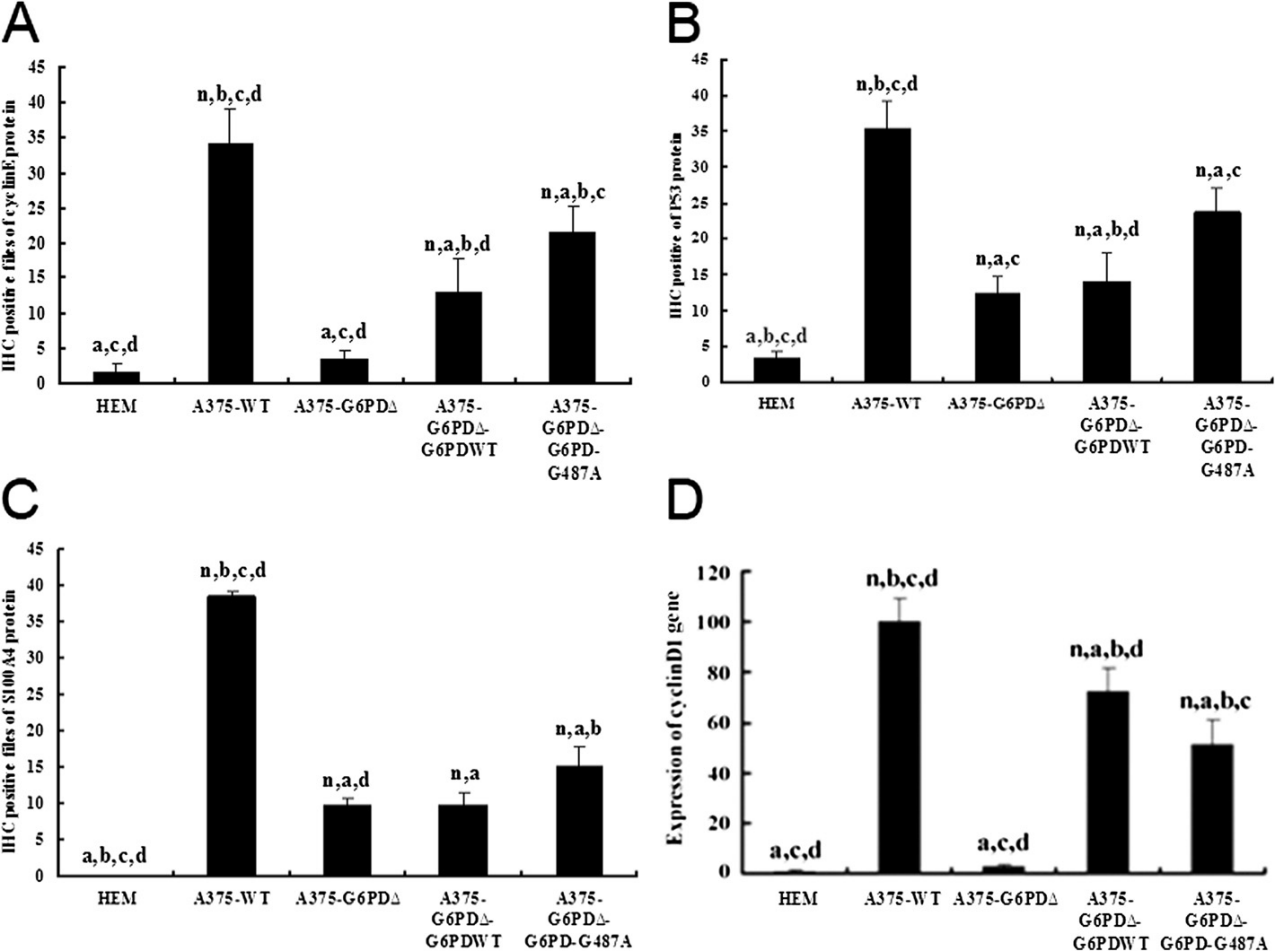
**Determination of cell cycle-related genes in tumor tissues of nude mice from different experimental groups. A**, amounts of cyclin E-positive cells in tumor tissues. **B**, amounts of p53-positive cells in tumor tissues. **C**, amounts of S100A4-positive cells in tumor tissues. n, a, b, c and d indicate statistically significant differences (*P* < 0.05), n vs. HEM; a vs. A375-WT; b vs. A375-G6PDΔ; c vs. A375-G6PDΔ-G6PDWT; d vs. A375-G6PDΔ- G6PD-G487A. **D**, mRNA expression of cyclinD1 detected by qRT-PCR.

The qRT-PCR showed that mRNA levels of cyclin D1 increased by 31-fold in the A375-WT cell group compared with the lowest observed levels in the A375-G6PD∆ cell group (*P* < 0.05, Figure 4D). Immunohistochemistry also suggested that the protein levels of Cyclin E, p53, and S100A4 in the A375-G6PD∆ group decreased by 77%, 60%, and 74%, respectively, compared with levels observed in the A375-WT group (Figure 4, Additional file 3: Figure S2, Additional file 4: Figure S3, Additional file 5: Figure S4). Both the mRNA level of cyclin D1 and the protein levels of these three genes in A375-G6PD∆-G6PD-WT and A375-G6PD∆-G6PD-G487A cell groups were determined to be partially recovered.

### Correlation of G6PD in tumor tissues with apoptosis-related protein expression

**Figure 5:**
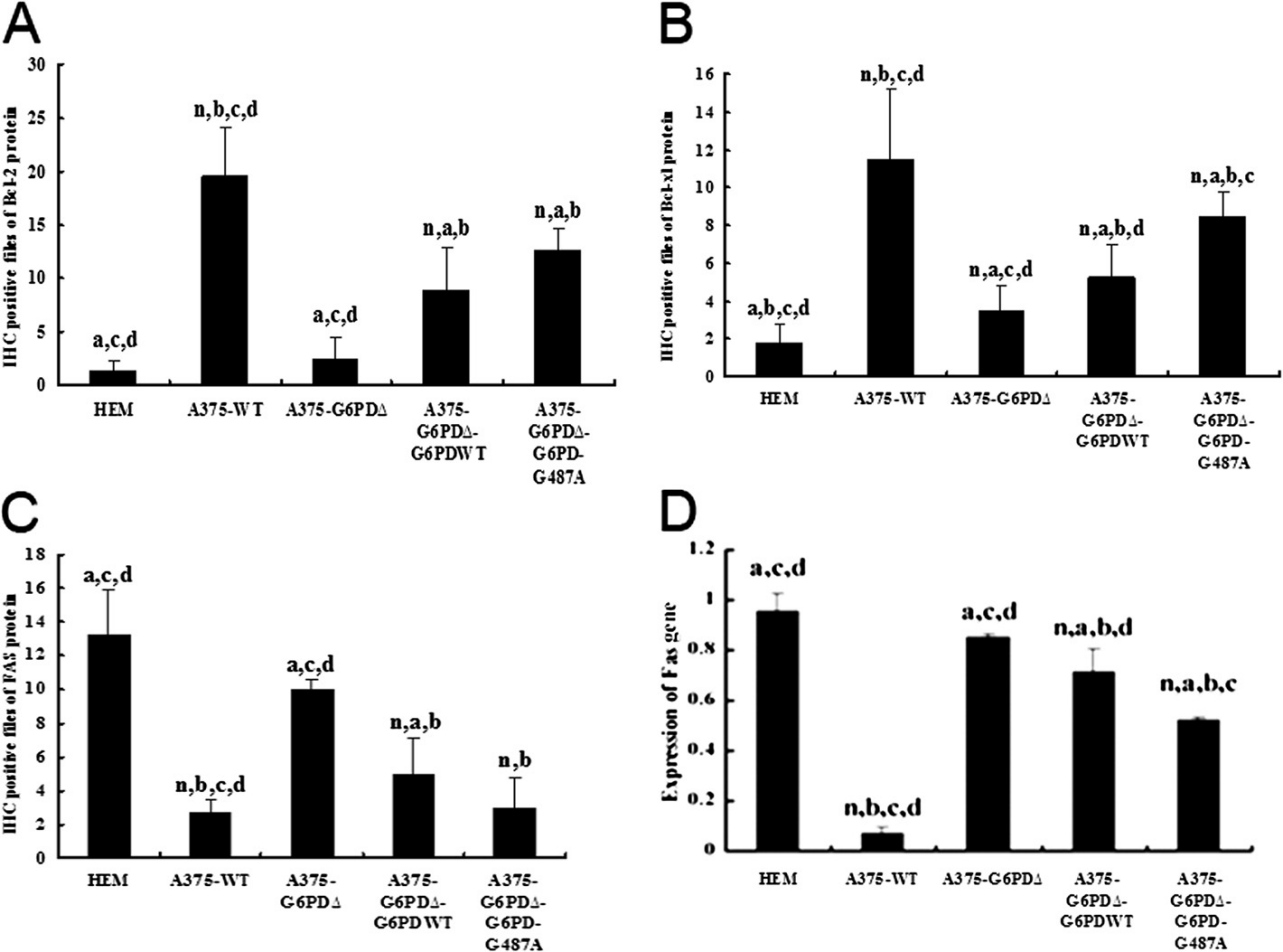
**Determination of cell apoptosis genes in tumor tissues of nude mice from different experimental groups. A**, amounts of Bcl-2-positive cells in tumor tissues **B**, amounts of Bcl-xl-positive cells in tumor tissues. **C**, amounts of Fas-positive cells in tumor tissues and n, a, b, c and d indicate statistically significant differences (*P* < 0.05), n vs. HEM; a vs. A375-WT; b vs. A375-G6PDΔ; c vs. A375-G6PDΔ-G6PDWT; d vs. A375-G6PDΔ- G6PD-G487A. **D**, mRNA expression of Fas detected by qRT-PCR.

Immunohistochemistry showed that the expression of apoptosis inhibitory factors Bcl-2 and Bcl-xL was significantly down-regulated in the A375-G6PD∆ cell group, while the expression of Fas protein increased by 72% compared with expression levels observed in the A375-WT cell group (Figure 5, Additional file 6: Figure S5, Additional file 7: Figure S6, Additional file 8: Figure S7). Protein expression data for Fas was also supported by the Fas mRNA levels detected by qRT-PCR (Figure 5D). Again, results in A375-G6PD∆-G6PD-WT and A375-G6PD∆-G6PD-G487A cell groups remained at moderate levels.

### Expression of *STAT3* and *STAT5* in tumor tissues

**Figure 6:**
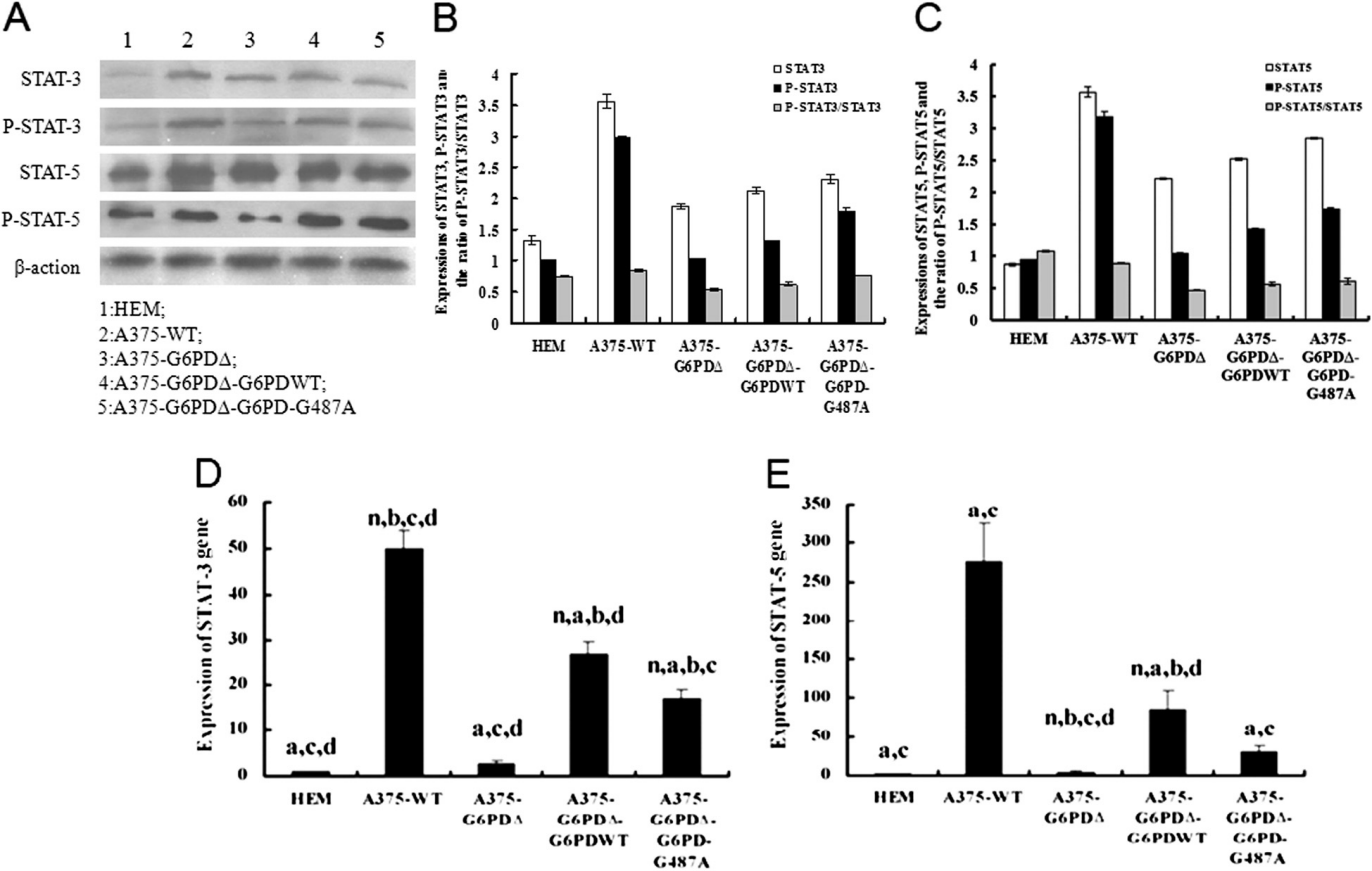
**Determination of STAT-3 and STAT-5 expression in tumor tissues.** Expression of STAT-3 and STAT-5 proteins and qualitative **(A)** and semi-quantitative analyses **(B** and **C)** of their phosphorylations. 1, HEM; 2, A375-WT; 3, A375-G6PDΔ; 4, A375-G6PDΔ-G6PDWT; 5, A375- G6PDΔ-G6PD-G487A. STAT3 is persistently activated in A375-WT cells. Compared with the HEM group, higher P-STAT3 expression is observed in the A375-WT cells group. Following knockdown of G6PD in wild-type A375 cells, the expression of both *STAT3* and *P-STAT3* decreases. The mRNA expression of STAT3 **(D)** and STAT5 **(E)**.

Western blot analysis results showed that STAT3, P-STAT3, STAT5, and P-STAT5 proteins decreased by 47%, 65%, 38%, and 67%, respectively, in the A375-G6PD∆ group as compared with results observed in the A375-WT group. Expressions of these proteins were observed to be partially restored in the A375-G6PD∆-G6PD-WT and A375-G6PD∆- G6PDG487A treated groups (Figure 6). The mRNA levels of STAT3 and STAT5 were detected by qRT-PCR, providing further support for these results (Figure 6D, 6E). Data presented in Figures 6B and 6C was generated from the average western blot data of all animal tissues in each group.

## Discussion

G6PD was previously shown to be highly expressed in human melanoma cells, exhibiting a close relationship to the growth and proliferative phenotype of tumor cells [24], although no *in vivo* study has confirmed this finding. Moreover, the underlying mechanism behind this correlation may be important in developing a complete understanding of the pathogenesis of melanoma and providing critical experimental evidence for the development of improved clinical treatment options. In the current study, as expected, expression and activity of G6PD showed a positive correlation with melanoma weight, growth, and differentiation. These findings suggest that *G6PD* expression in the A375 cell line plays an important role in tumor growth and proliferation. These findings are consistent with previous reports noting elevated expression or activity of G6PD in tumor cells [11-18]. Furthermore, expression and activity of G6PD was shown to be positively correlated with mRNA and protein expression of cyclins D1 and E, p53, and S100A4. These findings suggest that G6PD can regulate cell cycle through its effect on these factors, thus indirectly regulating melanoma growth.

Cyclin D1 accelerates the G1/S phase transition and promotes cell growth and division. Cyclin D1 overexpression is considered to be an important factor in the promotion of tumor occurrence and development, where it is also associated with reduced response to growth factors [27,28]. Elevated cyclin D1 gene expression in melanoma cells revealed that cyclin D1 was the key protein promoter of A375 cell proliferation and its overexpression can cause cell proliferation out of control which promotes tumor malignancy. Cyclin E is synthesized initially in the G1 phase, and expression increases in mid-G1 phase. Thereafter, cyclin E expression sharply increases and reaches its peak at the G1/S junction, followed by rapid disappearance in the S phase [29]. Immunohistochemical staining revealed few cyclin E-positive cells in the HEM group and significantly elevated cyclin E expression in the A375-WT group, suggesting that cyclin E is an important factor in melanoma occurrence and consistent with report by Bales E [30]. Furthermore, expression levels of cyclin D1 and E are positively correlated with expression of G6PD and its activity. These findings have indicated the vital regulation role of G6PD in uncontrolled cell proliferation and resultant malignant transformation in melanomas.

The tumor suppressor protein p53, a crucial regulator of the cell cycle in multicellular organisms, is also not expressed in certain cancers, including skin melanomas. It is, however, overexpressed in invasive melanomas with a high proliferative index [31,32]. Elevated p53 expression observed in A375 cells correlated well with high G6PD activity, suggesting that protein p53 is promoted by G6PD, which is consistent with study by Murtas et al. [33]. Jiang P found that the protein p53 affect the growth and invasion of melanoma cells in nude mice by interfering with the pentose-phosphate pathway in tumor cells [34]. These results suggest p53 may affect glucose metabolism in human melanoma when the activity of G6PD is interfered. S100A4 is a protein closely related to cellular differentiation and tumor occurrence, metastasis, and prognosis. S100A4 binds to the p53 protein and restricts the function of the G-S restriction point in the cell cycle. It may also be associated with uncontrolled entry in the M phase by bypassing the G2-M restriction point [35,36]. The present study showed that the level of S100A4-positive cells correlated well with p53, G6PD activity, and tumor growth, suggesting that G6PD affects p53 activity, thus also impacting melanoma occurrence and metastasis by regulation of S100A4 expression.

G6PD also showed a positive correlation to mRNA and protein expression of cell apoptosis inhibitory factors Bcl-2 and Bcl-xl and a negative correlation to Fas. Fas can be used as a marker to escape apoptosis by malignant cells. The immune escape mechanism employed by malignant melanomas involves actively attacking cytotoxic T lymphocytes (CTL) that express Fas to escape from apoptosis [37]. The Bcl-2 protein family affects apoptosis by regulating mitochondrial and endoplasmic reticulum stress-induced apoptotic pathways [38,39]. The proportion of apoptosis-inhibitory protein (Bcl-2) and apoptosis-promoting protein (Bax) determines the rate of apoptosis occurrence if cells are stimulated by apoptotic signals. Higher Bax/Bcl-2 ratios result in relatively higher sensitivity to CD95/Fas-mediated apoptosis [40]. In the present study, lower Fas expression in the A375-WT group and elevated Fas expression in the A375-G6PD∆ group with the G6PD knockout corresponded to low expression levels of apoptosis-inhibitory proteins Bcl-2 and Bcl-xL in the A375-G6PD∆ group. Similarly, high expression was observed in the A375-WT group, suggesting that G6PD may regulate cell apoptosis of melanoma through apoptosis-related factors Fas, Bcl-2, and Bcl-xL. Cumulatively, G6PD has a varied mechanism of action, affecting the levels of numerous other proteins associated with cell cycle regulation, thus promoting the development of malignancies.

The STAT pathway is well-down for its association with proliferation of malignant melanoma, escape from apoptotic signals, tumor invasion and angiogenesis [41]. More importantly, activation of STAT is the key factor for development of melanoma [42]; silencing of STAT3 using short hairpin RNA (shRNA) inhibits the growth of melanoma in tumor-bearing mice [43].

*STAT3* can down-regulate *Bcl-xL* expression and induce apoptosis in human melanoma A2058 and Jw cell lines [44]. In metastatic melanoma, the expression of phosphorylated *STAT3* is positively correlated with *Bcl-xL* expression [45]. Suppression of STAT5 protein in human melanoma A375 cells significantly down-regulates *Bcl-2* expression [46], while activation of STAT5 up-regulates *Bcl-xL* expression [42]. This indicates that STAT5 is an important anti-apoptotic factor [22,46]. Similarly, the present study indicated that *STAT3* and *STAT5* were positively correlated with Fas expression and negatively correlated with expression of *Bcl-2* and *Bcl-xL*. Leslie *et al*. [47] demonstrated that *Cyclin D1* mRNA levels were significantly increased in tumor cell lines that had excessive activation of the STAT3 signaling pathway, while mutagenesis of STAT3 binding sites within the cyclin D1 promoter region significantly inhibited the transcription of the *Cyclin D1* gene. *Cyclin D1* level was also positively correlated with STAT3 level in our study. Altogether, it suggests that the STAT3/STAT5 pathway could possibly be involved in the mechanism behind cell cycle abnormalities and cell apoptosis regulation.Further mechanistic studies, however, will be required to verify this mechanism.

## Conclusion

G6PD is involved in the growth, proliferation, and apoptosis of neoplastic tumors in nude mice models of melanoma. Furthermore, the tumor malignancy was observed to be in accord with G6PD protein expression. Higher expression of G6PD protein promoted the survival and proliferation of neoplastic tumors in nude mice models of melanoma (A375 cells) through the up-regulation of cyclin D1 and cyclin E, P53 and S100A4 protein expression. In this model, a deficiency of G6PD proteins in A375-G6PD∆ cells promoted cell apoptosis through down-regulation of the expressions of Bcl-2 and Bcl-xL and up-regulations of the expression of Fas. The correlation of G6PD expression and tumor growth corresponded with STAT3/STAT5 expression, although further experiments will be required to confirm the direct regulatory role of G6PD on this pathway. Because of the broad affects of G6PD, it may provide an excellent target for the development of improved treatment methods and prognostic indicators for melanoma patients.

## Competing interests

The authors declare that they have no competing interests.

## Authors’ contributions

TH and ZZ carried out the immunoassays. CZ and QT carried out real-time PCR and Western blot assay. BL setup the nude mice model. LC and TCcarried out the G6PD activity assay. YS and YZ carried out the design of the study and drafted the manuscript. All authors read and approved the final manuscript.

## Pre-publication history

The pre-publication history for this paper can be accessed here:

http://www.biomedcentral.com/1471-2407/13/251/prepub

## Supplementary Material

Additional file 1: Table S1 Pathological observations of neoplasm tumor in nude bearing melanoma model after injection of five types of cells (showed by HE staining). Click here for file

Additional file 2: Figure S1 HE staining of tumor tissues produced by injection of 5 types of cells. Click here for file

Additional file 3: Figure S2 Immunohistochemical staining of cyclin E protein in tumors formed by injection of 4 types of cells. Click here for file

Additional file 4: Figure S3 Immunohistochemical staining of p53 protein in tumors produced by injection of 4 types of cells. Click here for file

Additional file 5: Figure S4 Immunohistochemical staining of S100A4 protein in tumors produced by injection of 4 types of cells. Click here for file

Additional file 6: Figure S5 Immunohistochemical staining of Fas protein in tumors produced by injection of 4 types of cells. Click here for file

Additional file 7: Figure S6 Immunohistochemical staining of Bcl-2 protein in tumors produced by injection of 4 types of cells. Click here for file

Additional file 8: Figure S7 Immunohistochemical staining of Bcl-xL protein in tumors produced by injection of 4 types of cells. Click here for file
