# Supplementary material for: Variant G6PD levels promote tumor cell proliferation or apoptosis via the STAT3/5 pathway in the human melanoma xenograft mouse model
Source: BMC Cancer. 2013 May 22;13:251. doi: 10.1186/1471-2407-13-251 (PMC3765728; doi:10.1186/1471-2407-13-251)
Supplement: Additional file 1: Table S1 — Pathological observations of neoplasm tumor in nude bearing melanoma model after injection of five types of cells (showed by HE staining). [file 1471-2407-13-251-S1.doc]

**Supplementary Table 1 Pathological observations of neoplasm tumor in nude bearing melanoma model after injection of five types of cells (showed by HE staining)**

| Group | Visual observation | Microscopic observation | | | |
| --- | --- | --- | --- | --- | --- |
|  |  | Cell arrangement | Cell morphology | Nuclear morphology | Presence of complicated tissue necrosis |
| HEM | Small black spots that bulge out from the surface of nude mice skin, with distinct boundary with the surrounding tissues | Normal epidermal cells, and Inside and granular pigment distributed outside the cells | Brown or dark brown granular pigment with different sizes and shapes | No nuclear atypia | None |
| A375-WT | Tumors bulge out from the surface of nude mice skin, appearing red brown, with indistinct boundary with the surrounding tissues | Cancer cells appear nest-like and alveolar arrangement | Cancer cells with unequal sizes, abundant cytoplasm, appearing like polygons and spindles | Large nucleus, nuclear atypia, and many pathologic mitosis | Degeneration of some cells complicated by irregular necrosis |
| A375-G6PDΔ | Tumors slightly bulge out from the surface of nude mice skin, appearing nodular shape, with distinct boundary | Cancer cells appear nest-like arrangement | Cancer cells with equal sizes, no melanin granules found | Few nuclear atypia | No tissue necrosis |
| A375-G6PDΔ-  G6PDWT | Tumors bulge out from the surface of nude mice skin, appearing gray red, with partial adhesion to the surrounding tissues | Cancer cells appear nest-like arrangement | Cancer cells with unequal sizes, appearing like polygons | Nuclear atypia and pathologic mitosis | No tissue necrosis |
| A375-G6PDΔ-  G6PDG487A | Tumors bulge out from the surface of nude mice skin, appearing red, with partial adhesion to the surrounding tissues and difficulty to be moved | Cancer cells appear nest-like arrangement | Cancer cells with unequal sizes, appearing like polygons | Nuclear atypia and common pathologic mitosis | No tissue necrosis |

**Supplementary figure legends**

**Fig.1 HE staining of tumor tissues produced by injection of 5 types of cells**

**Fig.2 Immunohistochemical staining of cyclin E protein in tumors formed by injection of 4 types of cells**

**Fig. 3 Immunohistochemical staining of p53 protein in tumors produced by injection of 4 types of cells**

**Fig. 4 Immunohistochemical staining of S100A4 protein in tumors produced by injection of 4 types of cells**

**Fig. 5 Immunohistochemical staining of Fas protein in tumors produced by injection of 4 types of cells**

**Fig. 6 Immunohistochemical staining of Bcl-2 protein in tumors produced by injection of 4 types of cells**

**Fig. 7 Immunohistochemical staining of Bcl-xL protein in tumors produced by injection of 4 types of cells**
