# Supplementary figures and images for: Variant G6PD levels promote tumor cell proliferation or apoptosis via the STAT3/5 pathway in the human melanoma xenograft mouse model
Source: BMC Cancer. 2013 May 22;13:251. doi: 10.1186/1471-2407-13-251 (PMC3765728; doi:10.1186/1471-2407-13-251)

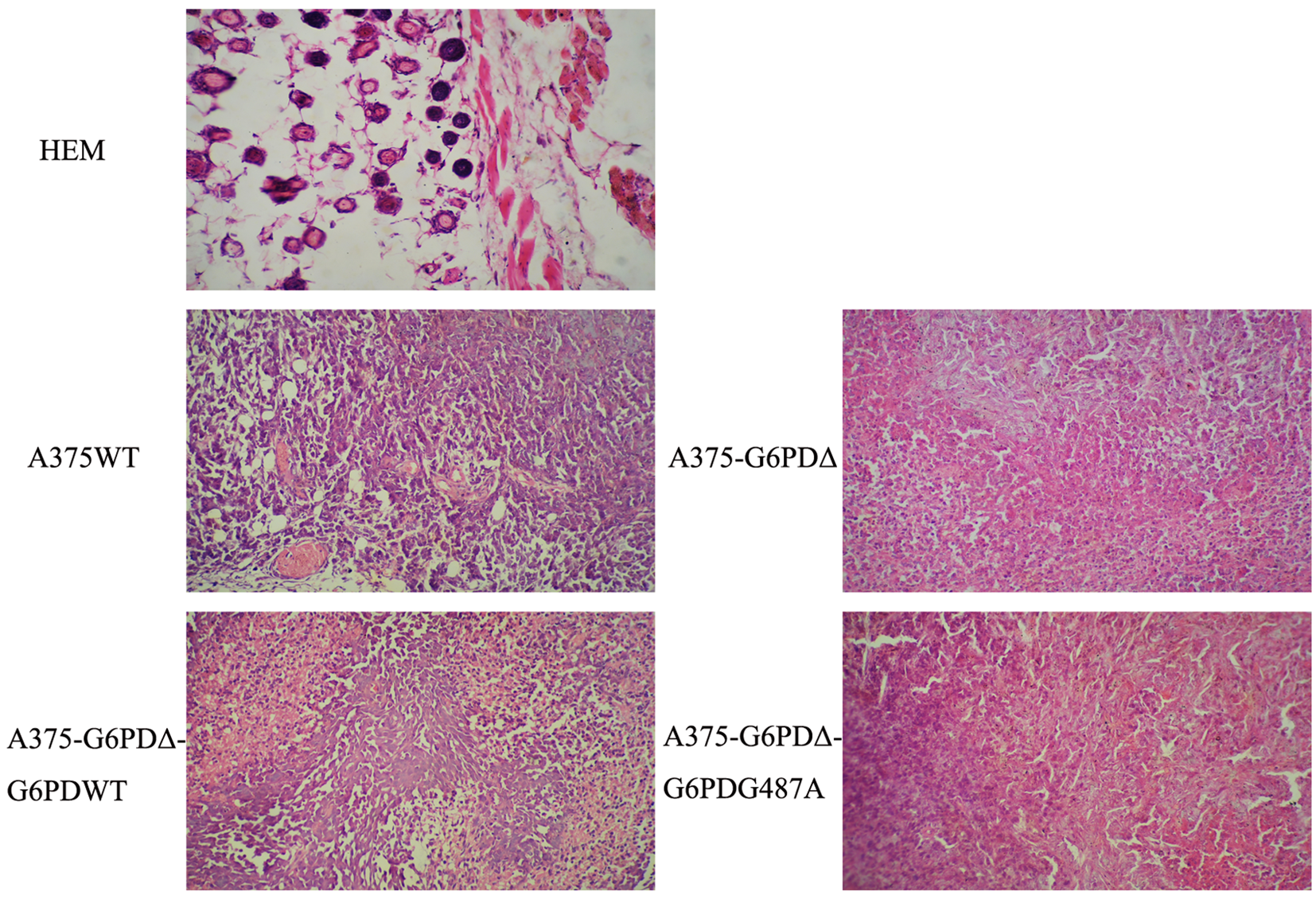

Supplement: Additional file 2: Figure S1 — HE staining of tumor tissues produced by injection of 5 types of cells. [file 1471-2407-13-251-S2.tiff]

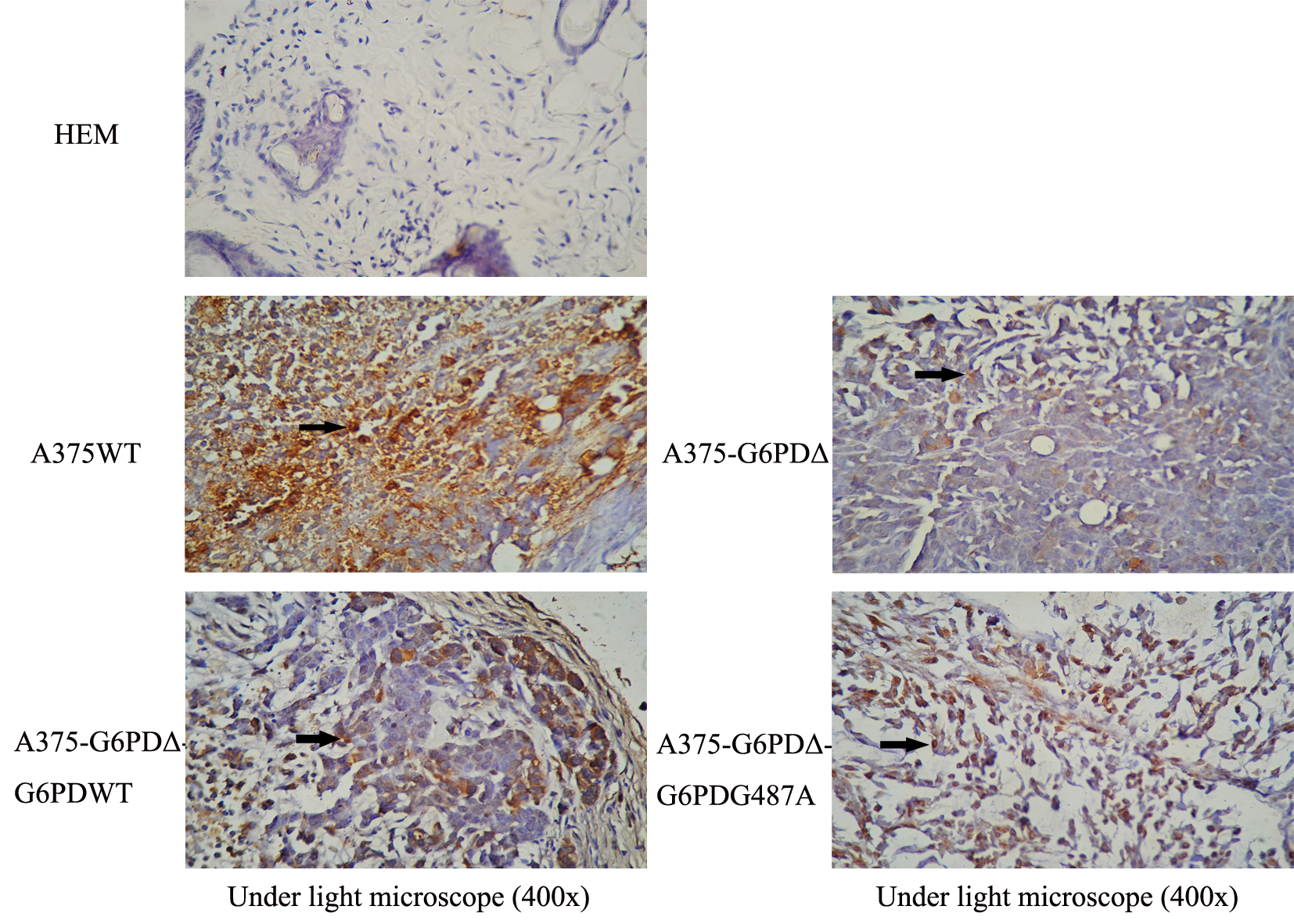

Supplement: Additional file 3: Figure S2 — Immunohistochemical staining of cyclin E protein in tumors formed by injection of 4 types of cells. [file 1471-2407-13-251-S3.tiff]

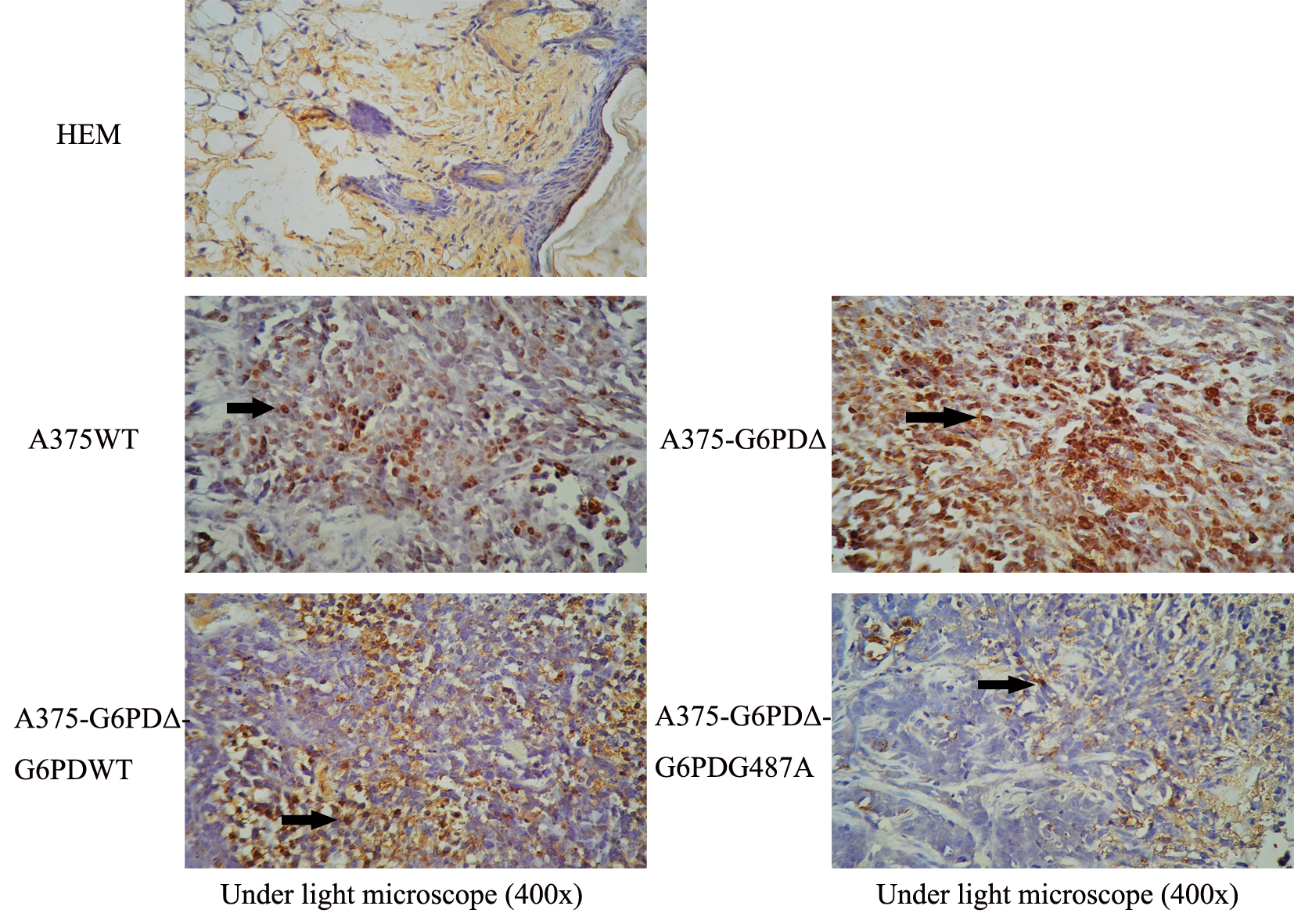

Supplement: Additional file 4: Figure S3 — Immunohistochemical staining of p53 protein in tumors produced by injection of 4 types of cells. [file 1471-2407-13-251-S4.tiff]

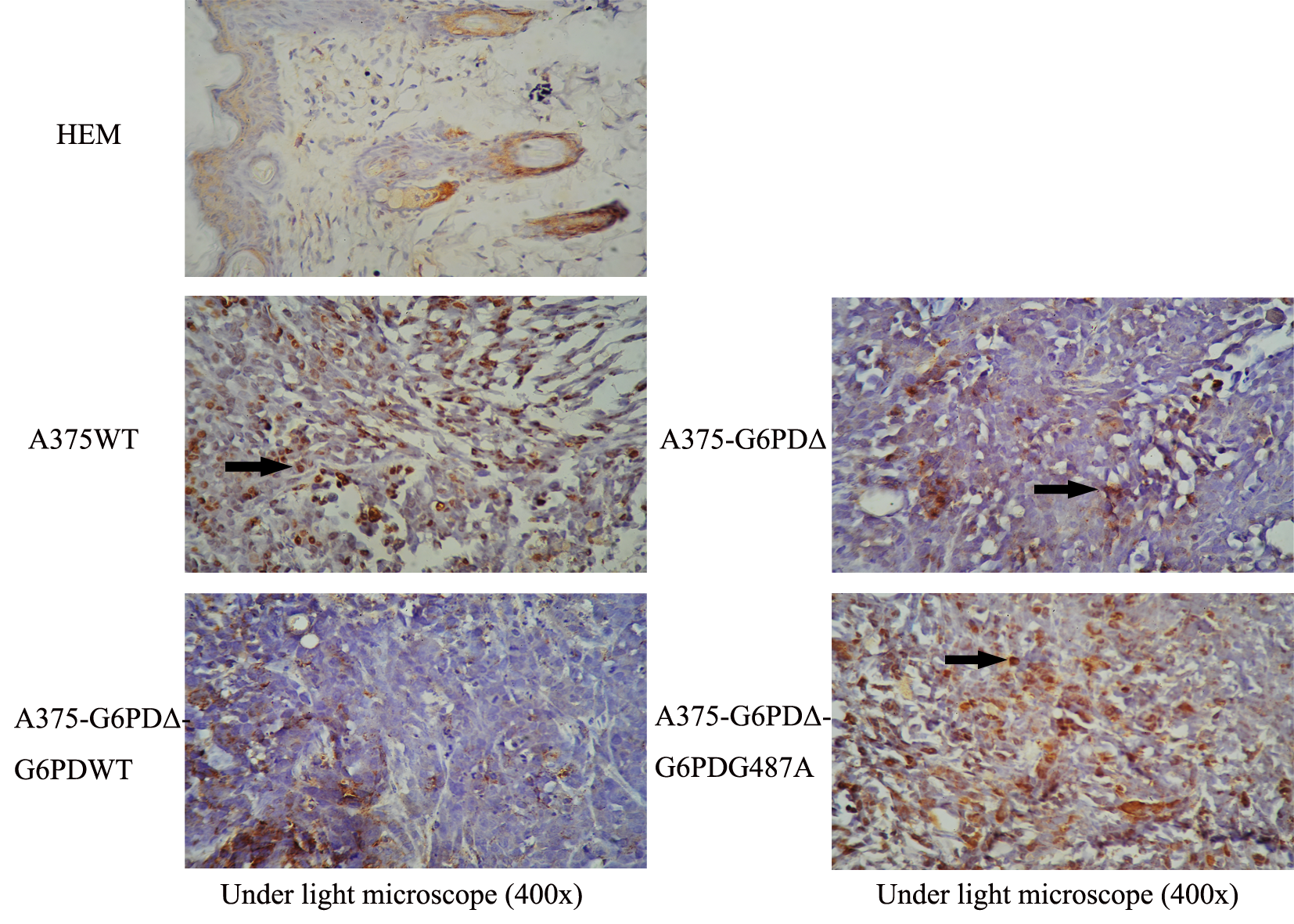

Supplement: Additional file 5: Figure S4 — Immunohistochemical staining of S100A4 protein in tumors produced by injection of 4 types of cells. [file 1471-2407-13-251-S5.tiff]

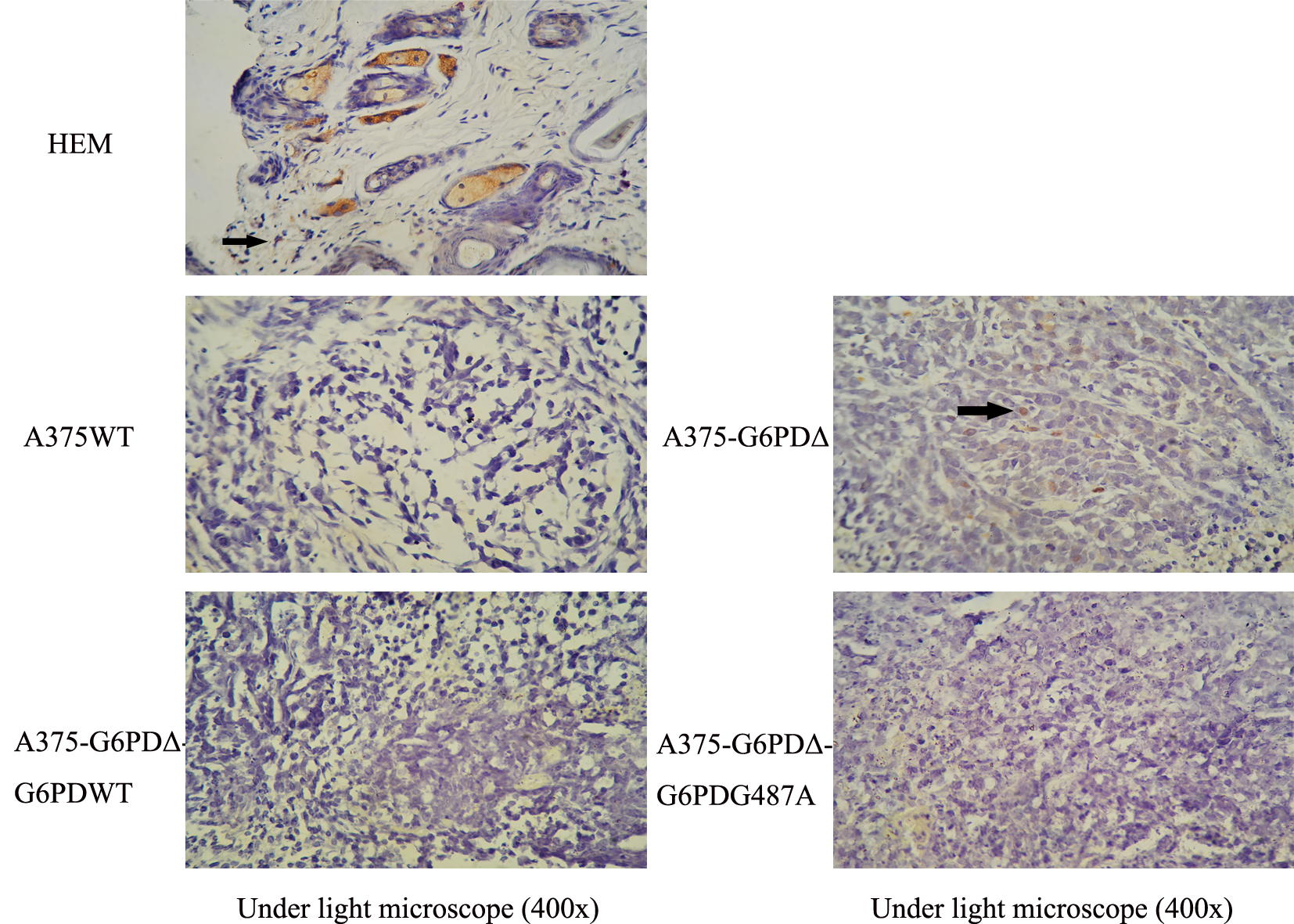

Supplement: Additional file 6: Figure S5 — Immunohistochemical staining of Fas protein in tumors produced by injection of 4 types of cells. [file 1471-2407-13-251-S6.tiff]

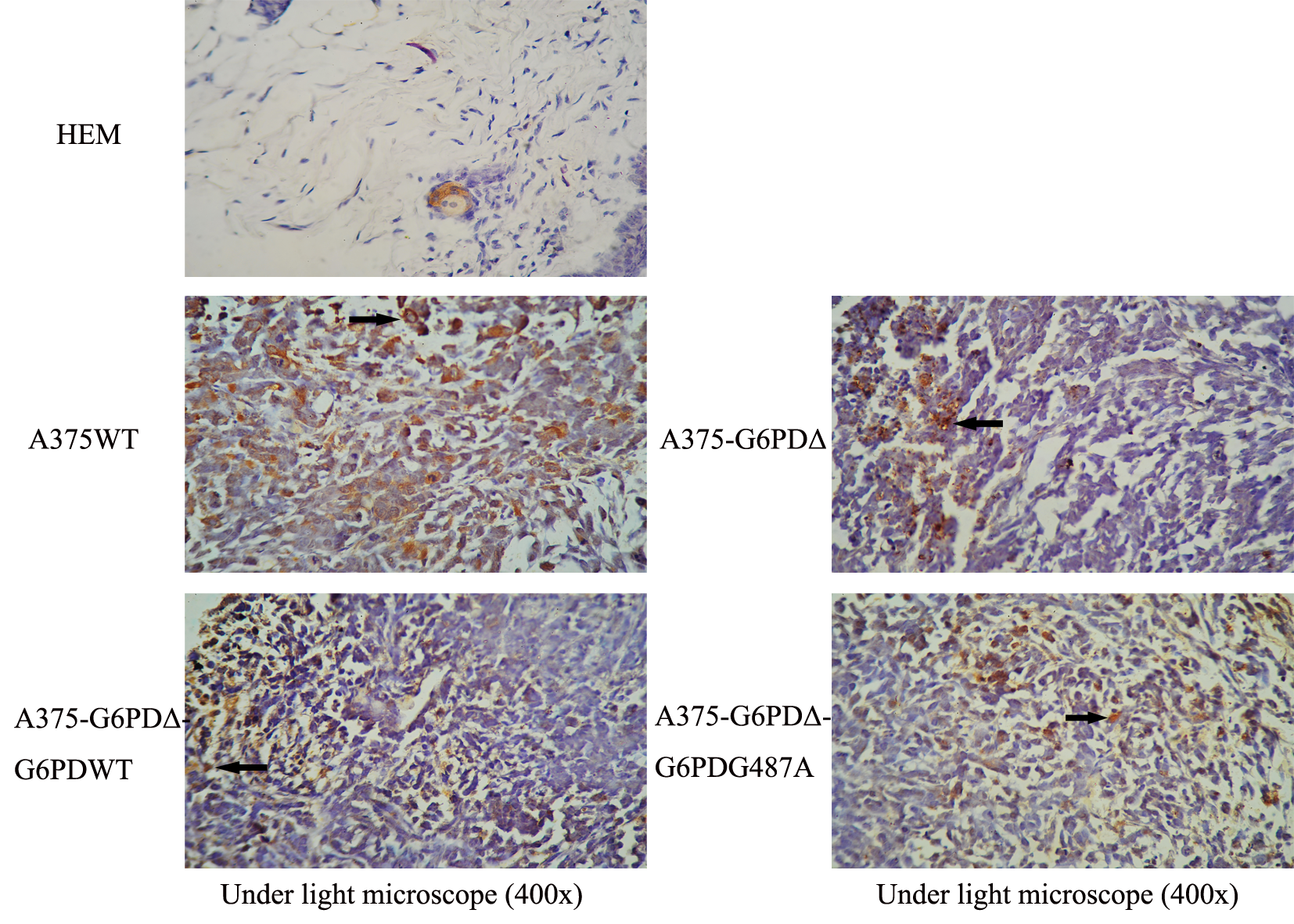

Supplement: Additional file 7: Figure S6 — Immunohistochemical staining of Bcl-2 protein in tumors produced by injection of 4 types of cells. [file 1471-2407-13-251-S7.tiff]

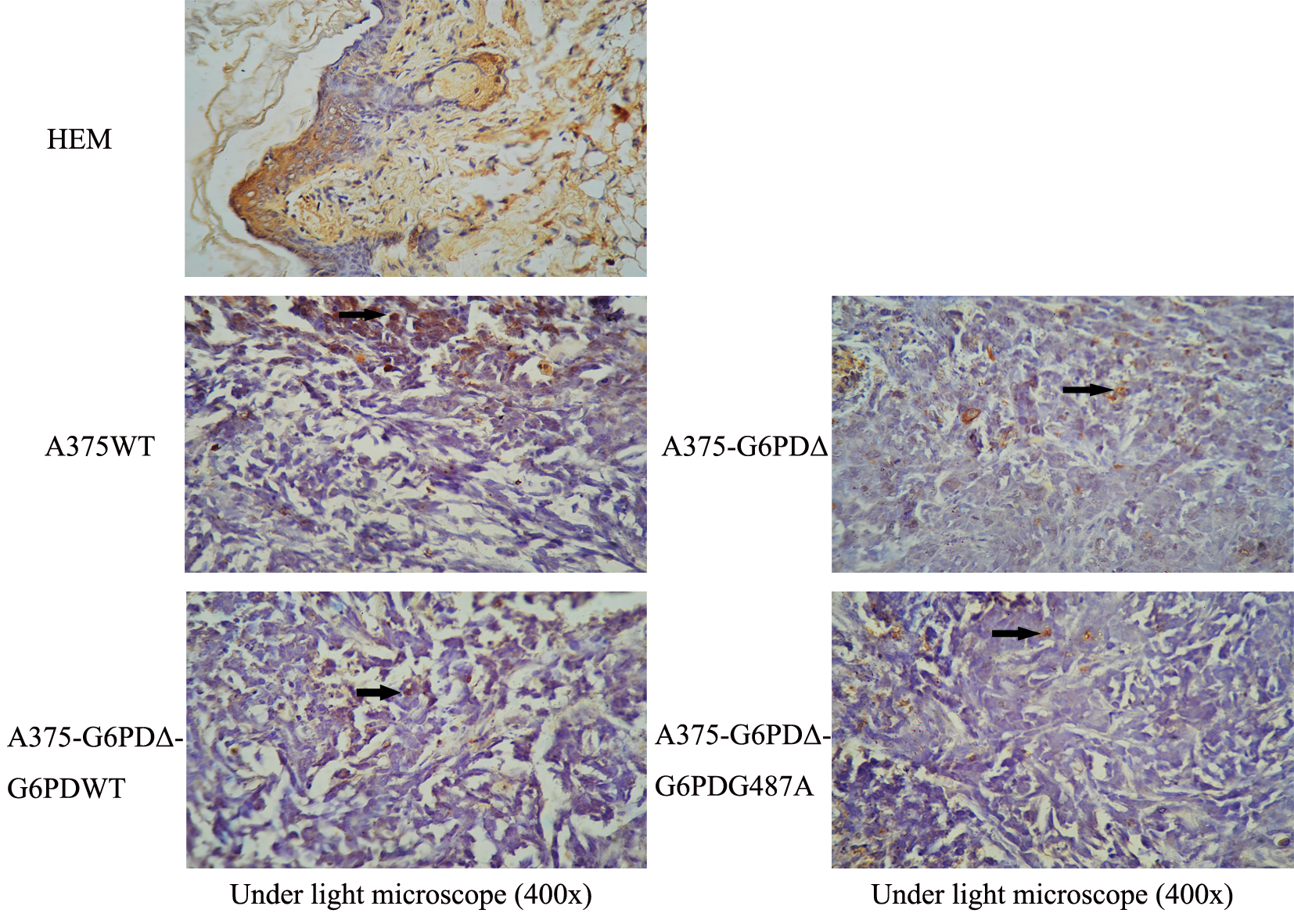

Supplement: Additional file 8: Figure S7 — Immunohistochemical staining of Bcl-xL protein in tumors produced by injection of 4 types of cells. [file 1471-2407-13-251-S8.tiff]
